# Thermal Nonlinear Klein–Gordon Equation for Nano-/Micro-Sized Metallic Particle–Attosecond Laser Pulse Interaction

**DOI:** 10.3390/ma14040857

**Published:** 2021-02-10

**Authors:** Mihai Oane, Muhammad Arif Mahmood, Andrei C. Popescu, Alexandra Bănică, Carmen Ristoscu, Ion N. Mihăilescu

**Affiliations:** 1 Electrons Accelerators Laboratory, National Institute for Laser, Plasma and Radiation Physics (INFLPR), 077125 Magurele-Ilfov, Romania; 2 Laser Department, National Institute for Laser, Plasma and Radiation Physics (INFLPR), 077125 Magurele-Ilfov, Romania; arif.mahmood@inflpr.ro (M.A.M.); alexandra.banica@inflpr.ro (A.B.); carmen.ristoscu@inflpr.ro (C.R.); ion.mihailescu@inflpr.ro (I.N.M.); 3 Faculty of Physics, University of Bucharest, 077125 Magurele-Ilfov, Romania; 4 Center for Advanced Laser Technologies (CETAL), National Institute for Laser, Plasma and Radiation Physics (INFLPR), 077125 Magurele-Ilfov, Romania; andrei.popescu@inflpr.ro; 5 Faculty of Medicine, Carol Davila University of Medicine and Pharmacy, 02002 Bucharest, Romania

**Keywords:** attosecond laser pulses, generalized Lambert–Beer law, Klein–Gordon heat equation in Kozłowski version, Au nano-/micro-particles, Zhukovsky mathematical formulation, ballistic versus thermal phenomena

## Abstract

In this study, a rigorous analytical solution to the thermal nonlinear Klein–Gordon equation in the Kozłowski version is provided. The Klein–Gordon heat equation is solved via the Zhukovsky “state-of-the-art” mathematical techniques. Our study can be regarded as an initial approximation of attosecond laser–particle interaction when the prevalent phenomenon is photon–electron interaction. The electrons interact with the laser beam, which means that the nucleus does not play a significant role in temperature distribution. The particle is supposed to be homogenous with respect to thermophysical properties. This theoretical approach could prove useful for the study of metallic nano-/micro-particles interacting with attosecond laser pulses. Specific applications for Au “nano” particles with a 50 nm radius and “micro” particles with 110, 130, 150, and 1000 nm radii under 100 attosecond laser pulse irradiation are considered. First, the cross-section is supposed to be proportional to the area of the particle, which is assumed to be a perfect sphere of radius R or a rotation ellipsoid. Second, the absorption coefficient is calculated using a semiclassical approach, taking into account the number of atoms per unit volume, the classical electron radius, the laser wavelength, and the atomic scattering factor (10 in case of Au), which cover all the basic aspects for the interaction between the attosecond laser and a nanoparticle. The model is applicable within the 100–2000 nm range. The main conclusion of the model is that for a range inferior to 1000 nm, a competition between ballistic and thermal phenomena occurs. For values in excess of 1000 nm, our study suggests that the thermal phenomena are dominant. Contrastingly, during the irradiation with fs pulses, this value is of the order of 100 nm. This theoretical model’s predictions could be soon confirmed with the new EU-ELI facilities in progress, which will generate pulses of 100 as at a 30 nm wavelength.

## 1. Introduction

During interactions between ultrashort laser pulses and metals, the heat transfer process can be observed in two steps. Firstly, energy absorption occurs via photon–electron interactions, followed by the return of excited electrons to the initial state in a few femtoseconds. Next, the energy is redistributed from the electrons to the lattice by electron–phonon interactions within a few picoseconds. Thermalization follows, i.e., the heat is dissipated, and the lattice reaches the thermal equilibrium [1,2,3]. For micro- and nano-particle interactions with ultrashort laser pulses, the mechanism is more intricate, particularly in the case of attosecond pulses, where the laser pulse duration is inferior to the electron–phonon relaxation time. To study this process, quantum theory must be applied. Nowadays, a wavelength with a value of 0.15 nm can be effectively reached. Attosecond optics is considered today a subcategory of ultrafast optics and strong-field physics.

There are very few well-established methods to solve the heat equation. For example, the integral transform technique was successfully applied ten years ago. The attempt to apply the mentioned formalism to the ultrashort scale (distance or time) did not provide impressive results. Hence, in this study, a novel, semiclassical technique is developed and applied for the interaction between nano-/micro-Au particles and ultrafast laser radiation.

## 2. Kozłowski Thermal Model

In the present section, we briefly refer to the Kozłowski thermal model, proposed in [4,5]. The study of transport mechanisms at the nano-/micro-scale is of huge importance nowadays. In particular, nanoparticles and nanotubes demonstrate critical physical applications for nanoscale heat transfer [6,7]. There exist a number of models developed in a simple manner to point-like particles. The progress of ultrashort pulses presents new opportunities to investigate the dynamics of electrons in nanoscale systems: carbon nanotubes and nanoparticles. In the case of attosecond laser pulses, the pulse duration is shorter than the electrons’ relaxation time. Consequently, transport equations consist of the second-order partial derivative concerning time. The nonlinear Klein–Gordon equation for mass and thermal energy transport at the nanoscale is considered in [4]. In [5], in the case of ultrashort laser pulses, heat transport is described by the hyperbolic heat transport equation (2-dimensional space (r,z)):

(1) $$\tau \frac{\partial^{2}T}{\partial t^{2}}+\frac{\partial T}{\partial t}=D\left(\frac{\partial^{2}T}{\partial r^{2}}+\frac{\partial^{2}T}{\partial z^{2}}\right)$$

T denotes the temperature variation of the electron gas in nanoparticles, τ is the atomic relaxation time, D is the thermal diffusivity, r is the radial component of the target, and z is the laser direction propagation normal to the surface of the radial component plane. R≤r≤R and −R≤z≤R can be obtained because the origin of spatial coordinates are placed in the center of the target for convenience. The relaxation time is defined as

(2) $$\tau =\frac{\hbar }{{\mathrm{mv}}^{2}}$$

where v is the thermal pulse propagation speed. In the case of relativistic electron scattering, c is the speed of light in a vacuum, while m is the electron mass.

Thus,

(3) $$\tau =\frac{\hbar }{{\mathrm{mc}}^{2}}$$

The number of electrons, N, inside a Au particle (a sphere with a radius R or a rotation ellipsoid) can be expressed as

(4) $$N=\frac{\frac{4{\pi R}^{3}}{3}}{\mu }\rho \mathrm{AZ}\propto R^{3}$$

(5) $$N=\frac{\frac{4\pi \mathrm{abc}}{3}}{\mu }\rho \mathrm{AZ}$$

where ρ is the density of nano-/micro-particles; A is the Avogadro number; µ is the molecular mass of the Au atoms in grams; a, b, and c are semiaxes for the ellipsoid shape of the particle; Z is the number of electrons in a single Au atom. It is worth mentioning that a, b, and c should be at least of the same order of magnitude; that is, the Au nano-/micro-particle contains N/Z Au atoms. According to the Kozłowski approach [4,5], the relaxation time τ for nano-/micro-particle consisting of N electrons is

(6) $$\tau^{N}\propto R^{3}\tau$$

The thermal relaxation process in nanoparticles, which implies N light scatters points, can be described on the basis of the “atomic” parameter τ.

The following “correspondences” are used from [5] to transform the classical heat equation to a quantum heat equation:

(7) $$\tau \to R^{3}\tau$$

(8) $$D\to \frac{\hbar }{m}$$

By applying Equations (7) and (8) to Equation (1), the heat equation becomes

(9) $$R^{3}\tau \frac{\partial^{2}T}{\partial t^{2}}+\frac{\partial T}{\partial t}=\frac{\hbar }{m}\left(\frac{\partial^{2}T}{\partial r^{2}}+\frac{\partial^{2}T}{\partial z^{2}}\right)$$

Equation (9) is the linear-damped Klein–Gordon equation, and for nanotechnological applications, it is solved using the nonlinear d’Alembert equation [4].

Our approach uses the Zhukovsky’s mathematical model to solve Equation (9). As a relevant example, calculations were made with Au particles. However, they can be easily converted to any other noble metal.

## 3. Zhukovsky Mathematical Model

The thermal Klein–Gordon equation in the Zhukovsky model [8,9] explicitly includes the heat and thermal wave equations. If Equation (1) is solved according to [9], the following solution (see Appendix A) can be obtained:

(10) $$T\left(r,z,t\right)={℮}^{\;-\;\frac{ℇt}{2}}\cdot \;\frac{t}{4\sqrt{\pi }}\overset{\infty }{\underset{0}{\int }}\frac{d\xi }{\xi \sqrt{\xi }}\;\cdot \;{℮}^{-\;\frac{t^{2}}{16\;\xi \;}-{\;\xi }^{2\;}\left({ℇ}^{2}\right)}{\hat{S}}_{r}{\hat{S}}_{Z}\;f\left(r,z\right)$$

where:

(11) $$f\left(r,z\right)\equiv T\;\left(r,z,0\right)$$

$f\left(r,z\right)$, defined as an initial temperature, serves in the Zhukovsky model as a source term. Consequently, the Zhukovsky model becomes more adequate the shorter the pulse irradiation time is. In Equation (10) ε=1/τ, and ξ is a real positive increment factor. The heat operator can be further defined as

(12) $${\hat{S}}_{i}=\;℮{\;}^{-4\frac{D}{\tau }\xi \partial_{i}^{2}}f\left(i\right),\;\mathrm{where}\;i=r,z.$$

## 4. The Generalized Lambert–Beer Law

Since we work with ultrashort laser pulses (which mostly affect the nanoparticle surface), and the target is small (in nm regime), it is reasonable to assume the following approximation for Equation (11) (see Appendix B)—the generalized Lambert–Beer law:

(13) $$f\left(r,z\right)=T\;\left(r,z,0\right)\propto I_{o}e^{-\alpha z}{\pi r}^{2}$$

To obtain Equation (13), we used the approximation in which the cross-section is proportional to the area of the nanoparticle [10], and we calculated the absorption coefficient via a semiclassical approach [11]. Here,$\;I_{o}$ is the laser beam intensity and α is the absorption coefficient. In this case, the electron temperature variation of a nano-/micro-particle under ultrashort laser pulse irradiation is [8,9]

(14) $$T\left(r,z,t\right)=e^{-\frac{t}{2\tau }}\frac{t}{4\sqrt{\pi }}\int_{0}^{\infty }\frac{d\xi }{\xi \sqrt{\xi }}e^{-\frac{t^{2}}{16\xi }-\frac{\xi }{\tau^{2}}}\hat{S_{r}}\hat{S_{z}}f\left(r,z\right)$$

It was taken into account that the spatial shape of the laser beam is a flat top-hat propagating along the *z*-axis. We have (Appendix A)

(15) $${\hat{S}}_{r}\left(r^{2}\right)=4r^{2}-2$$

and

(16) $${\hat{S}}_{z}e^{-\alpha z}=e^{-\alpha z}$$

The final formula for the electrons temperature is a result of Equations (12)–(16):

(17) $$T\left(r,z,t\right)=I_{0}\cdot e^{-\frac{t}{2\tau }}\cdot \frac{t\cdot \left(4r^{2}-2\right)}{(4\sqrt{\pi )}}\cdot \overset{\infty }{\underset{0}{\int }}d\xi /(\xi \sqrt{\xi })\cdot e^{\frac{-t^{2}}{16\xi }\;-\;\frac{\xi }{\tau^{2}}}\cdot e^{-4\cdot \alpha \cdot z\;\cdot \frac{D}{\tau }\cdot \xi }$$

It should be noted that ξ is the increment from Equation (12), and τ is given by Equation (7). α is the absorption coefficient calculated in Appendix B. The absorption coefficient of a Au particle irradiated by a 100 as laser pulse is [11]

(18) $$\alpha \left(\lambda \right)=N\sigma \left(\lambda \right)=N2r_{e}{\lambda f}_{2}$$

where N is the number of atoms per unit volume, r_e_ is the classical electron radius (2.82 × 10^−15^ m), and λ is the laser wavelength (30 nm), while f_2_ is an atomic scattering factor for Au [12]. Irradiation with a single pulse is considered, which is a reasonable assumption [11].

## 5. Simulations Based on the Thermal Klein–Gordon Equation

The simulations were conducted for Au nanoparticles with a 50 nm radius, as well as for microparticles with 110, 130, 150, and 1000 nm radii, respectively, submitted to 100 as laser pulse irradiation under the liquid (water). This regime is currently described in the literature as pulsed laser irradiation under liquid (PLIL) [13]. The modeling can be easily extended to other materials under different liquid environments by appropriately changing the calculation parameters. Water was chosen as an environment because its heat transfer coefficient is suitable for studying gold nano- and micro-particles’ thermal properties under laser irradiation [13]. Indeed, the water heat transfer coefficient allows the avoidance of the thermal runaway and preserves the interaction process’s actual thermal properties. We developed a theoretical model different from the usual ones [14,15,16].

**Figure 1:**
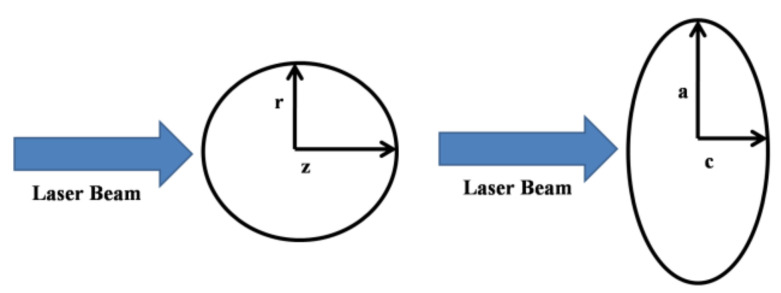
Geometry of the computational method: a sphere versus a rotation ellipsoid.

Figure 1 schematically depicts the geometry of our studies. The laser beam propagates along the z-axis, and the center of the coordinate’s axes coincides with the center of the irradiated configuration.

**Figure 2:**
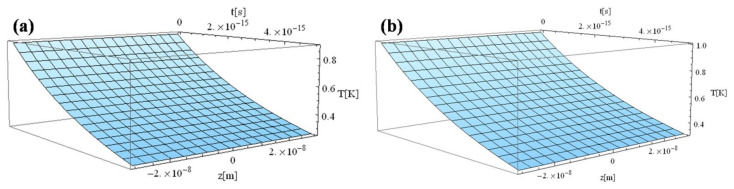
Electron temperature versus time and z during 1 fs simulation after irradiation with a 100 as laser pulse applied to an Au nanoparticle: (**a**) 40 × 20 × 30 nm^3^ rotation ellipsoid; (**b**) 30 nm radius sphere.

Figure 2 shows a comparison of electron temperature in the case of an ellipsoid and a spherical Au nanoparticle, respectively. It can be seen that the ellipsoid electron temperature is inferior to that of the spherical particle. This is due to the fact that the ellipsoid interaction area (800 nm^2^) is smaller than the spherical area (900 nm^2^).

The following figures, Figure 3, Figure 4, Figure 5, Figure 6 and Figure 7, correspond to spherical nanoparticles of different sizes, while Figure 8, Figure 9 and Figure 10 were computed for different simulation times.

**Figure 3:**
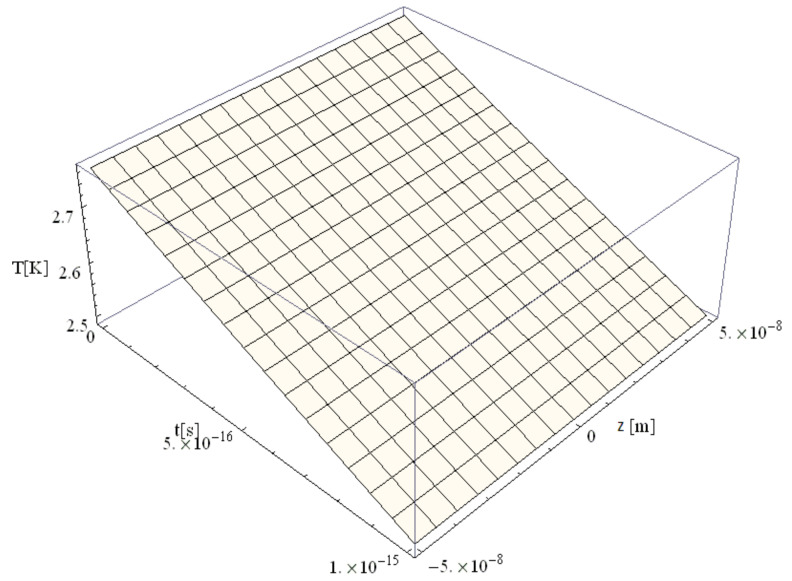
Electron temperature versus time and z during 1 fs simulation after irradiation with a 100 as laser pulse applied to an Au nanoparticle with a 50 nm radius.

The temperature field is presented in Figure 3 for a simulation time of 1 fs simulation after irradiation with a 100 as laser pulse applied to a Au nanoparticle with a 50 nm radius. The temperature gradient is minimal (≤2.8 K), which leads to the conclusion that ballistic phenomena still play a significant role.

**Figure 4:**
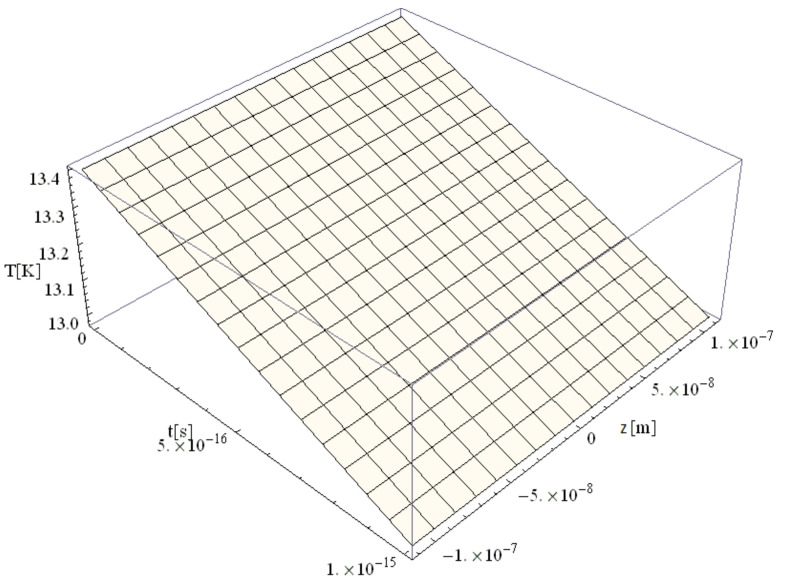
Electron temperature versus time and z during 1 fs simulation after irradiation with a 100 as laser pulse applied to an Au microparticle with a 110 nm radius.

**Figure 5:**
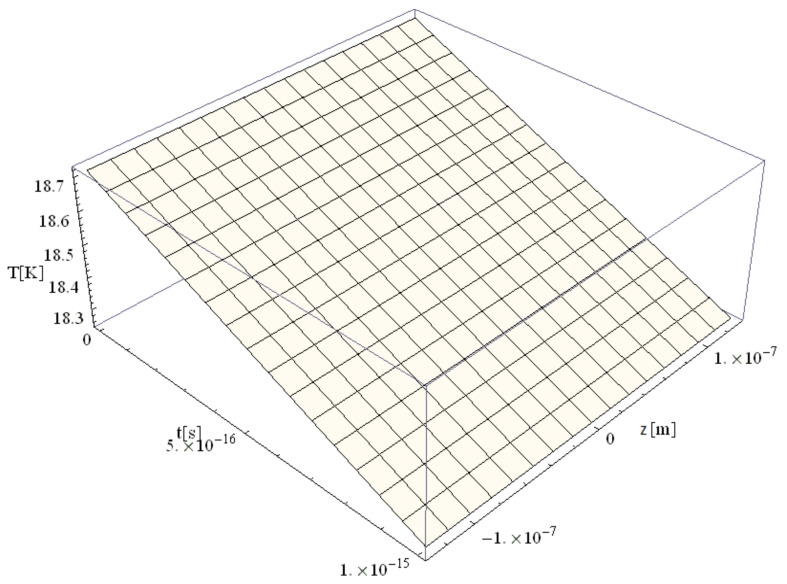
Electron temperature versus time and z during 1 fs simulation after irradiation with a 100 as laser pulse applied to an Au microparticle with a 130 nm radius.

**Figure 6:**
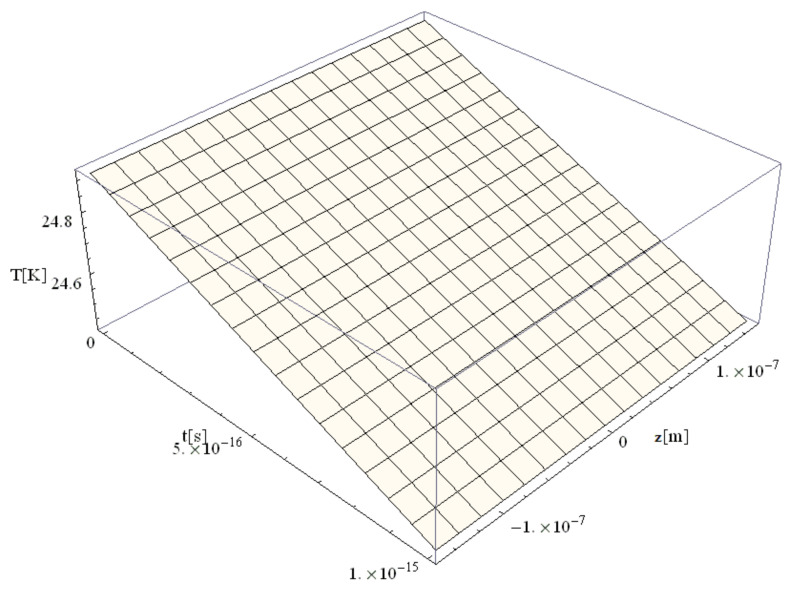
Electron temperature versus time and z during 1 fs simulation after irradiation with a 100 as laser pulse applied to an Au microparticle with a 150 nm radius.

**Figure 7:**
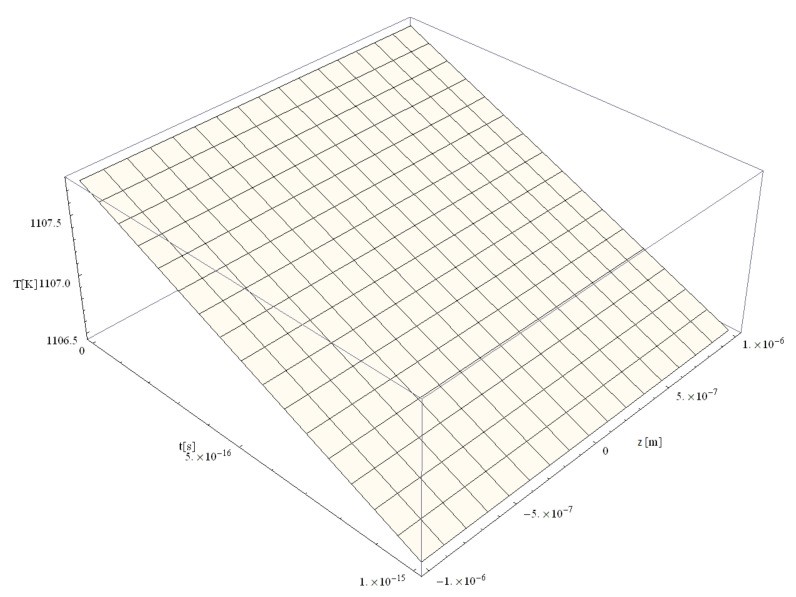
Electron temperature versus time and z during 1 fs simulation after irradiation with a 100 as laser pulse applied to an Au microparticle with a 1000 nm radius.

Figure 4, Figure 5, Figure 6 and Figure 7 show the variation of the electron temperature during the 1 fs simulation of Au microparticles with radii of 110, 130, 150, and 1000 nm, respectively, after irradiation with a 100 as laser pulse.

According to Figure 4, Figure 5, Figure 6 and Figure 7, the particle size (radius) plays a crucial role in determining the temperature variation in the volume of the spherical particle: the larger the radius is, the higher the electron temperature variation results. This is because the laser beam–particle interaction is proportional to the geometrical cross-section of the particle:$\;\sigma \;\sim {\;\pi R}^{2}\;$[10].

From Figure 4, Figure 5, Figure 6 and Figure 7, it can be observed that the real transitions from ballistic to thermal behavior takes place between 260 nm (Figure 6) and 2000 nm (Figure 7).

The electron temperatures in Figure 4, Figure 5, Figure 6 and Figure 7 are consistently superior to those in Figure 3. They are ≤13.4 K for a 110 nm radius, ≤18.7 K for a 130 nm radius, and ≤24.8 K for a 150 nm radius, eventually becoming ≤1108 K for a 1000 nm radius. The jump is of one order of magnitude in the first three cases, rising to about 500 times in the case of the 1000 nm radius. This, in our opinion, is the effect of the significant decrease in the curvature degree and is in full accordance with the modification from nano to micro status (at about 50 nm radius, i.e., a 100 nm diameter), in the last four cases as compared to the first ones (Figure 3). On the other hand, the estimated maximum temperature (1108 K) stays inferior to the Au melting point (1337 K). This warrants the application of the Klein–Gordon equation in the Kozłovski–Zhukovsky form in our simulations when no phase transition from solid to liquid is induced.

One may conclude that the Au particle size overwhelmingly determines the temperature variation (increase) inside the target volume to be irradiated to a more considerable extent in the micro case than in the nano case.

**Figure 8:**
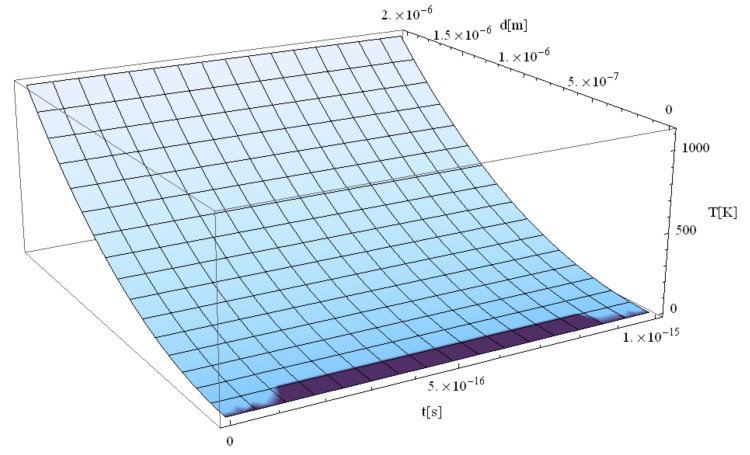
Electron temperature versus radius during 1 fs simulation after irradiation with a 100 as laser pulse applied to an Au particle.

**Figure 9:**
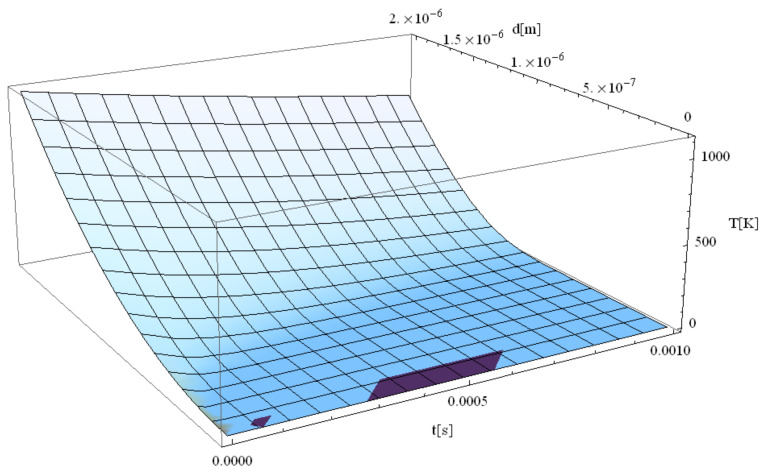
Electron temperature versus radius during 1 ms simulation after irradiation with a 100 as laser pulse applied to an Au particle.

**Figure 10:**
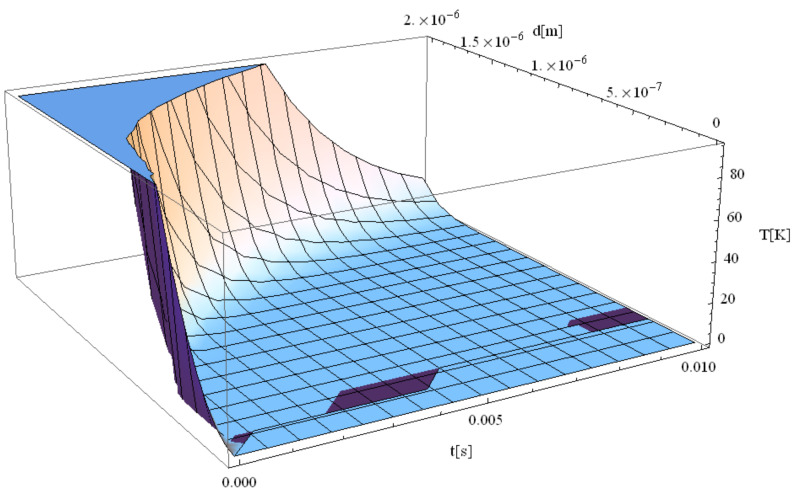
Electron temperature versus radius during 0.01 s simulation after irradiation with a 100-laser pulse applied to an Au particle.

Next, we studied the correlation between the Au particle electron temperature field interacting with the laser and the increasing computation time. The results are given in Figure 8, Figure 9, Figure 10 for simulation times of 1 fs, 1 ms, and 10 ms, respectively. A drastic decrease in temperature with the increase in the time range can be observed.

## 6. Conclusion

This study combines two “state-of the-art” issues and provides a powerful model of nano-/micro-particle heating and attosecond laser pulse irradiation.

1. For demonstration, the analysis was conducted for Au particles in the nano (100 nm) to micro (220–2000) nm range. A computational system with the following specifications was used to plot electron temperature profiles: core i7, 4th generation, 16 Gb Ram. The electron temperature graphics were generated after 1 min of simulation.
2. The results show that the electron temperature variation strongly depends on particle size, both in nano- and micro-regimes. Thus, the larger the particle size, the larger the maximum temperature value spreading inside the particle.
3. Longer simulation times (a few to tens of fs) allowed for a more accurate thermal field prediction after a longer thermalization time.
4. The simulations were conducted for nanoparticles under 100 attosecond pulse laser irradiation. We attempted to develop a coherent approach using (i) the Kozłowski theoretical models [4,5] to take into account quantum effects, (ii) the Zhukovsky mathematical apparatus [8,9] to be able to consider the minimal time of irradiation (100 as), and (iii) the Zavestovskaya–Kanavin hypothesis to generalize the Lambert–Beer law as close to reality as possible [10].
5. The main physical conclusion at the nanoscale is that we observed a dominant ballistic phenomenon, while for values higher than 500 nm, the two mechanisms (ballistic and thermal) compete. The shorter the target and irradiation time, the higher the presence of ballistic phenomena [17]. Compared to fs-scale irradiation, where the two phenomena are present at 100 nm [18], the same behavior within the range of 500–1000 nm can be observed. Our study suggests that the thermal field becomes dominant in the range exceeding 1000 nm.

## Data Availability

Data sharing is not applicable.

## Appendix A

Using the mathematical approach in [9] and considering $\hat{S}$ the heat operator, $e^{\gamma x}x^{k}$ the source term, v a parameter from generalized hyperbolic heat equation (set at 0, to be in concordance with our heat equation), H the Hermite function of two variables, and r the radius of nano-/micro-particle [9], the following can be obtained:

(A1) $${\hat{S}e}^{\gamma x}x^{k}=e^{\eta \partial_{x}^{2}}x^{k}e^{\gamma x}=e^{\gamma x+\gamma^{2}v}H_{k}\left(x+2\gamma v,v\right),k\;\epsilon \;\mathrm{Integers},\;\gamma \;\epsilon \;\mathrm{Reals},$$

(A2) $$\left\{\begin{array}{c} \gamma =0\\ k=2 \end{array}\right.$$

(A3) $${\hat{S}\;r}^{2}=H_{2}\left(r,v\right)=4r^{2}-2+4v$$

(A4) $$\left\{\begin{array}{c} \gamma =-\alpha \\ k=0 \end{array}\right.$$

and

(A5) $${\hat{S}\;e}^{-\alpha z}=e^{-\alpha z+\alpha^{2}v}H_{0}\left(z,0\right);{\;H}_{0}\left(z,0\right)=1$$

Equation (A1) explains the generalized working of heat operators, which was solved for two particular cases when (a) γ=0 and k=2, and (b) γ=−α and k=0 with the solutions provided in Equations (A3) and (A5), respectively.

## Appendix B

The absorption coefficient of a Au particle irradiated by a 100 as laser pulse is as follows [11]:

(A6) $$\alpha \left(\lambda \right)=N\sigma \left(\lambda \right)=N2r_{e}{\lambda f}_{2}$$

where N is the number of atoms per unit volume, *r_e_* is the classical electron radius ($2.82\times 10^{-15}\;m$), λ is the laser wavelength (30 nm), and f_2_ is an atomic scattering factor for Au. Since the photon energy for the 30 nm wavelength is 41.3608 eV, it can be assumed that f_2_ = 10 [12]. Following the calculations, $\alpha =\;20.196\;\left(mm^{-1}\right)$ can be otained.
